# Young, but not in the dark—The influence of reduced lighting on gait stability in middle-aged adults

**DOI:** 10.1371/journal.pone.0280535

**Published:** 2023-05-18

**Authors:** Tirtsa Naaman, Roee Hayek, Itai Gutman, Shmuel Springer

**Affiliations:** Faculty of Health Sciences, Department of Physical Therapy, The Neuromuscular & Human Performance Laboratory, Ariel University, Ariel, Israel; Kennedy Krieger Institute/Johns Hopkins University School of Medicine, UNITED STATES

## Abstract

**Background:**

The aim of this study was to investigate the effects of walking in reduced lighting with or without performing a secondary cognitive task on gait dynamics in middle-aged adults and to compare them with young and old adults.

**Methods:**

Twenty young (age 28.8±4.1), 20 middle-aged (age 50.2±4.4), and 19 elderly (age 70.7±4.2) subjects participated in the study. Subjects walked on an instrumented treadmill at a self-determined pace under four conditions in randomized order: (1) walking in usual lighting (1000 lumens); (2) walking in near-darkness (5 lumens); (3) walking in usual lighting with a serial-7 subtraction dual-task; and (4) walking in near-darkness with a serial-7 subtraction dual-task. Variability in stride time and variability in the trajectory of the center of pressure in the sagittal and frontal planes (anterior/posterior and lateral variability) were measured. Repeated measures ANOVA and planned comparisons were used to determine the effects of age, lighting conditions, and cognitive task on each gait outcome.

**Results:**

Under usual lighting, stride time variability and anterior/posterior variability of the middle-aged subjects were similar to those of the young and lower than those of the old. The lateral variability of the middle-aged subjects was higher than that of young adults under both lighting conditions. Similar to the older adults, the middle-aged participants increased their stride time variability when walking in near-darkness, but they were the only ones to exhibit increased lateral variability and anterior/posterior variability in near-darkness. Young adult gait was not affected by lighting, and concurrent performance of a cognitive task while walking did not affect gait stability in all groups under any of the lighting conditions.

**Conclusions:**

Gait stability decreases in middle age when walking in the dark. Recognition of functional deficits in middle age could promote appropriate interventions to optimize aging and reduce fall risk.

## Introduction

Falls resulting from deterioration of gait stability are a major public health problem. Stable human locomotion requires coordination between somatosensory input and visual information that governs foot position control. The important role of vision in gait regulation has been well described [1]. Online visual information is critical for successfully negotiating obstacles or walking over complex terrain [2–5], and a sudden transition from normal to marginal illumination challenges movement control [6]. Reliance on visual information to maintain postural stability increases with age, and impaired visual perception due to a reduction in light is associated with high fall risk [7–9]. The ability to walk in reduced lighting conditions during aging is substantial for safe navigation in everyday environments and may help prevent falls; therefore, it may affect quality of life [10].

Most studies that have examined the effects of limited light intensity on walking have tested the variability of gait pattern, as it represents the ability to optimally control gait from one stride to the next and is considered a reliable way to quantify balance during locomotion [11]. Figueiro et al. [12] reported increased step length variability when older adults walked on a dim walkway. Kesler and colleagues [13] demonstrated that older adults with high levels gait disorders exhibit increased stride and swing time variability when lighting was reduced, and even healthy older adults experienced a slowing of gait speed. In healthy young adults, deprivation of visual information during treadmill walking increased step width variability, but stride time and stride length increased only when subjects walked slower than their preferred walking speed [14]. Another study that investigated the effect of visual deprivation (i.e., closed eyes) on dynamic stability showed no age-related differences in gait variability between young and older adults while walking on a treadmill [15]. Other measures of gait dynamics during treadmill walking, such as frontal plane trunk acceleration variability, did not differ when healthy adults aged 44±14 years walked with open or closed eyes [16].

Cohort-based population studies show that the annual prevalence of falls triples from 9% in 40- to 44-year-old adults to 28% in 60- to 64-year-old adults [17,18] suggesting that falls are not only a problem of old age. Gait variability has also been shown to increase at an accelerated rate at middle age, which may be a relevant early indicator of fall risk [19–21]. These results support the premise that middle age might be a critical life stage for identifying indicators for fall risk. It has been previously shown that increasing gait complexity may help to identify changes in balance in middle-aged adults [22].

Despite the increased prevalence of falls in middle age, the effects of reduced visual input on gait stability in middle-aged adults have rarely been investigated. Furthermore, many of the currently available references examined the effects of visual deprivation on dynamic stability while walking with eyes closed [14–16]. However, it has been shown that walking with eyes closed can be easier than walking with eyes open without visual cues [23]. Finally, assessing gait variability under challenging situations such as walking while performing a secondary cognitive task can also provide information about postural control during walking. Many studies have demonstrated a significant interaction between cognitive domains and walking control [24–26]. In addition, the ability to walk while performing other tasks (e.g., talking on a smartphone while walking) is important for maintaining many daily activities [27].

Therefore, the aim of this study was to investigate the effects of reduced lighting during walking with or without a dual task on gait variability in middle-aged individuals and to compare their response with that of young and old adults. To better describe our cohort, we also examined physical performance and activity, as these have been associated with general health and cognitive function [28,29].

## Methods

### Participants

The study included a convenience sample of 20 young adults (age 28.8±4.1), 20 middle aged adults (age 50.2±4.4), and 19 older adults (age70.7±4.2).

Participants were included if they lived in the community, were independent in activities of daily living, and could walk without assistance. Subjects with neurological, orthopedic, vestibular, or visual impairments (e.g., age-related macular degeneration, glaucoma, cataract, diabetic retinopathy) or other comorbidities that could affect gait were excluded. The study was approved by Ariel University Ethics Committee (approval number AU-HEA-SS-20210809). All subjects gave written informed consent to participate in the study.

### Procedure

Each subject participated in a single session that lasted approximately 60 minutes.

Before assessing gait, anthropometric measures were collected, physical performance was quantified using handgrip strength (Jamar®, 5030J1, Patterson Medical, Warrenville, IL, USA) and the 30-second chair-stand test (30CST) [30], and physical activity was measured using the Beacke Physical Activity Questionnaire (BPAQ) [31].

Gait was assessed while the subjects walk over an instrumented treadmill (Zebris FDM-T; Zebris Medical GmbH, Isny, Germany) in a self-paced speed under four conditions in randomized order: (1) walking in usual lighting (1000 lumens); (2) walking in near-darkness (5 lumens); (3) walking in usual lighting with dual-task; and (4) walking in near-darkness with dual-task. Each gait trail lasted 3 min, with 3 min rest between tests. During the dual-task conditions, subjects walked while reciting out loud serial subtractions of 7, starting from a different 3-digit number at each trial. No instructions regarding priority of walking vs. cognitive task were given. Before performing the dual task while walking, the task was measured for 120 s while sitting, to examine the ability of the subjects to perform an arithmetic task the score of this test was based on number of correct responses (Serial-7 sitting). In addition, subjects were given the opportunity to become accustomed to walking on a treadmill in usual lighting for 6–7 minutes [32]. During the familiarization period gait speed was gradually increased and the maximum comfortable walking speed achieved by each subject was used for all gait tests. For safety reasons, subjects wore a harness (without body weight support) that did not impede movement of the arms and legs.

The Zebris instrumented treadmill consists of a capacitance-based foot pressure platform in the treadmill with a sensing area of 101.6 × 47.4 cm, containing 6720 sensors measuring at 240 Hz. The treadmill’s dedicated software (FDM V1.18.48) provides data on spatiotemporal gait parameters and center of pressure (COP) trajectories during walking. The following gait outcomes were derived from the treadmill software [33]: (1). Stride time variability: the standard deviation of stride times divided by the average stride time; (2). Anterior/posterior variability: the standard deviation of the intersection point of the CoP trajectory on the frontal axis; (3). Lateral variability: the standard deviation of the intersection point of the CoP trajectory on the transverse axis. The anterior/posterior and lateral variability were derived from the dedicated treadmill software. The software generates a graphical pattern representing a continuous trace of the CoP trajectory during walking, with "zero’ deviations from the CoP intercepts corresponding to constant strides.

### Statistical analysis

Shapiro-Wilk test verified that all variables were normally distributed. Background variables (i.e., height, weight, serial-7 sitting, hand grip strength, 30CST, BPAQ, and treadmill walking speed) were compared between groups using ANOVA followed by post hoc analyses. A 3 X 2 X 2 repeated measure ANOVA and planned comparisons were used to determine the effect age group (young, middle age, Old) X lighting condition (usual lighting, near-darkness) X dual task (with/without dual task) on each gait outcome. An additional ANOVA examined the effect of age group on serial-7 task performance during walking (serial-7 walking). Partial η2 effect sizes were calculated for each ANOVA model, with η2 = 0.01 indicating a small effect, η2 = 0.06 indicating a medium effect, and η2 = 0.14 indicating a large effect [34]. Statistical analysis was performed using IBM SPSS Statistics, version 27.0. (Armonk, NY: IBM Corp), and significance was set at p<0.05.

## Results

### Background variables

*Table 1* summarizes the background characteristics of the participants. There were significant differences between groups in maximal grip strength (p = 0.002, partial η2 = 0.182), ability to move from sitting to standing (p<0.001, partial η2 = 0.347), and self-selected treadmill walking speed (p = 0.003, partial η2 = 0.185). To test the possibility that these variables are covariates in the main analysis, we analyzed the correlation between all three variables and age. The correlations of each variable with age were larger and more significant than all other correlations. Therefore, age was considered as a mediating factor between these three variables and the dependent variables (i.e., gait outcomes). The older adults had slower self-selected treadmill walking speed and lower grip strength compared to the middle-aged (p = 0.004, and p = 0.018 respectively) and the young adults (p = 0.028, and p = 0.005, respectively); however, there were no significant differences in these parameters between the middle-aged and young groups. The young adults had a better ability to move from sitting to standing than the middle-aged adults (p = 0.005) and old adults (p<0.001), while there was no significant difference between the middle-aged and old groups.

**Table 1:** Participant characteristics.

	Young (N = 20)	Middle age (N = 20)	Old (N = 19)	p-value
**Gender: Female (%)**	10 (50%)	10 (50%)	10 (52%)	0.986
**Height (cm)**	171.5±11.7	170.1±11.1	168.4±7.8	0.645
**Weight (Kg)**	70.4±15.3	68.6±15.4	70.3±10.5	0.905
**BMI**	23.7±3.4	23.4±3.4	24.8±3.3	0.425
**BPAQ**	7.3±2.4	6.8 ±1.7	6.2 ±1.3	0.213
**Serial-7 sitting**	21.4±9.2	19.6±10.9	14.2±8.8	0.064
**Maximal grip**^**a**^ **(KgF/Kg)**	0.56±0.14	0.53±0.12	0.41±0.15*^#^	0.002
**CST30 (N)**	27.6 ±6.7^**#**^	21.6 ±5.6*	17.6 ±4.7*	<0.001
**Treadmill walking speed**^**b**^ **(m/sec)**	0.63±0.1	0.60±0.13	0.50±0.11*^#^	0.003

Values are presented as mean ±SD.

BMI:Body mass index; CTS30:Chair-stand test 30 seconds; BPAQ:Beacke physical activity questionnaire.

^a^ normalized to body weight

^b^ normalized to height.

*significant difference compared to young.

^#^significant difference compared to middle age.

### Gait variability

The gait variability results can be found in Appendix 1 in S1 File.

#### Stride time variability

A significant effect on stride time variability was found for age (p<0.001, partial η^2^ = 0.482) and lighting condition (p<0.001, partial η^2^ = 0.523), but not for dual-task (p = 0.289). In addition, a significant interaction was found between the effects of age and lighting condition (p = 0.018, partial η^2^ = 0.128).

The older group had significantly higher stride time variability than the middle-aged and young groups (p<0.001 in both comparisons), but there was no difference in stride time variability between the middle-aged group and the young group (p = 0.441). The difference in stride time variability between the old and middle-aged groups was smaller in near-darkness than in usual lighting.

**Fig 1:**
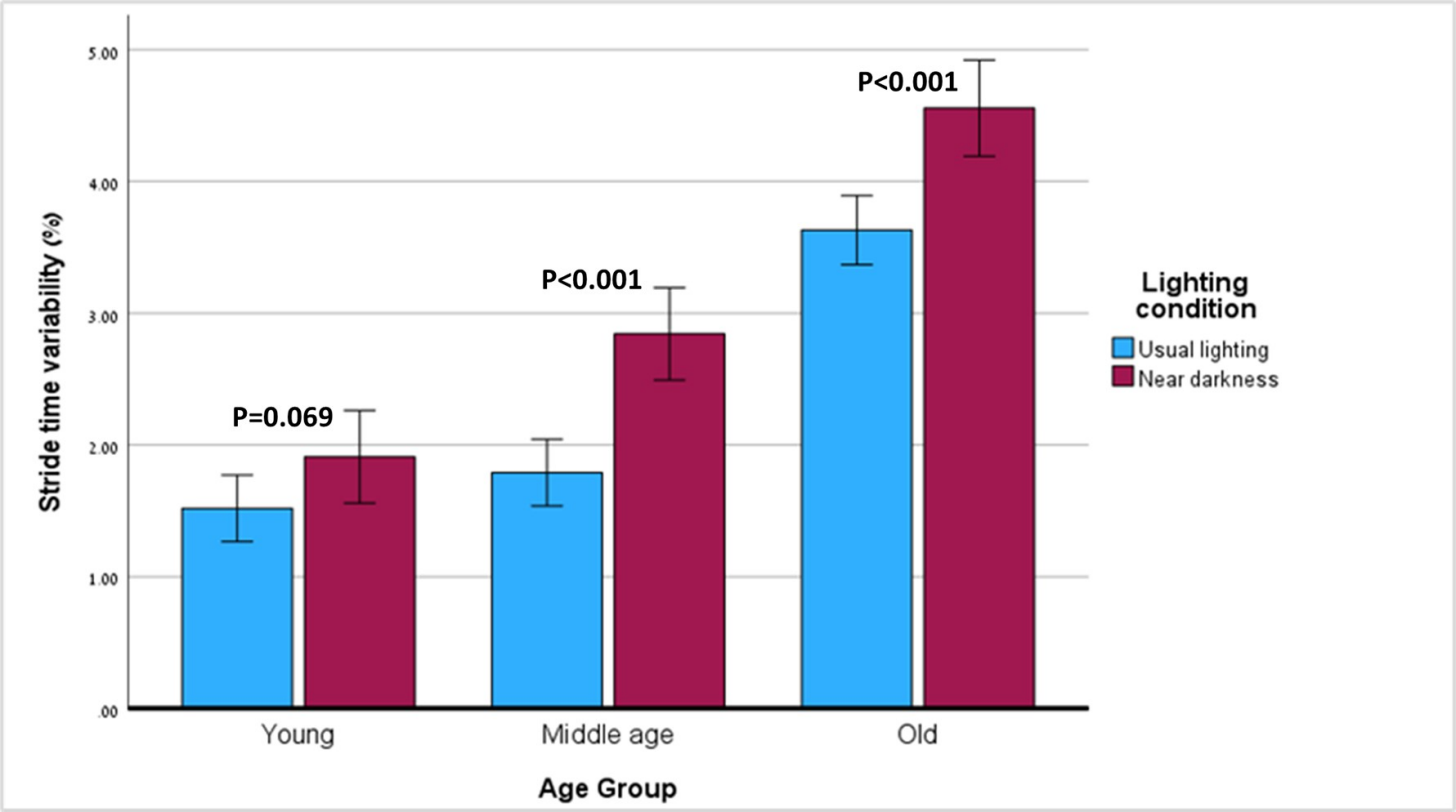
Stride time variability in the two lighting conditions—within-group comparisons.

Within-group comparisons of stride time variability in the three groups under the two lighting conditions is shown in Fig 1. The old and middle-aged groups increased their stride time variability in near-dark compared to usual lighting (p<0.001 in both comparisons, partial η^2^ = 0.496 and 0.732 respectively), whereas the young adults’ stride time variability did not change (p = 0.069).

#### Anterior/Posterior variability

A significant effect on anterior/posterior variability was found for age (p<0.001, partial η^2^ = 0.319) and lighting condition (p = 0.007, partial η^2^ = 0.143), but not for dual-task (p = 0.561). There was no interaction between the different effects. To further investigate the effect of near darkness, our between-groups analysis showed a significant difference in anterior/posterior variability between the old group and the young group (6.37, 95% CI: 5.59–7.15 vs. 3.953, 95% CI: 3.194–4.712, p<0.001) and between the old group and the middle-aged group (6.37 vs. 4.36, 95% CI: 3.55–5.16, p = 0.002), but not between the middle-aged group and the young group (p = 1.000).

**Fig 2:**
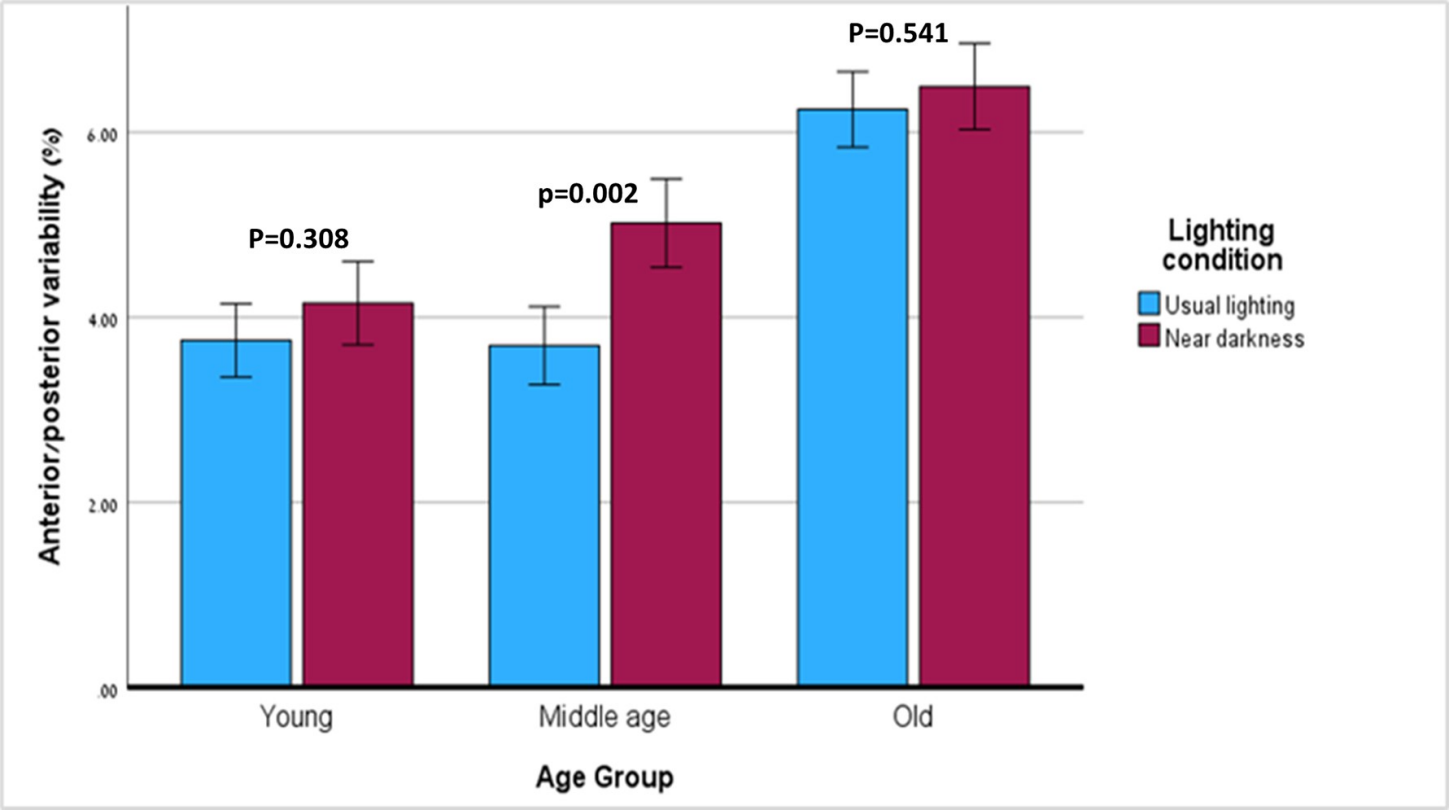
Anterior/Posterior variability in the two lighting conditions—within-group comparisons.

Within-group comparisons of anterior/posterior variability in the three groups under the two lighting conditions is shown in Fig 2. The middle-aged group had increased anterior/posterior variability in near-dark compared to usual lighting (3.69, 95% CI: 2.85–4.54 in usual light versus 5.02, 95% CI: 4.05–5.98in near dark, p = 0.002, partial η^2^ = 0.657). In contrast, the young and the old groups did not change their anterior/posterior variability (p = 0.308, p = 0.541, respectively).

#### Lateral variability

A significant effect on lateral variability was found for age (p<0.001, partial η^2^ = 0.427) and lighting condition (p = 0.004, partial η^2^ = 0.157), but not for dual-task (p = 0.204). Also, a significant interaction was demonstrated between the effects of age group and lighting condition (p = 0.009, partial η^2^ = 0.177). The old group had increased lateral variability compared to the middle-aged group (11.58 vs. 7.11, 95% CI: 5.21–9.02, p = 0.005) and the young group (11.58, 95% CI: 9.67–133.48 vs. 3.59, 95% CI: 1.74–5.44, p<0.001). The middle-aged group had increased lateral variability compared to the young group (p = 0.031).

**Fig 3:**
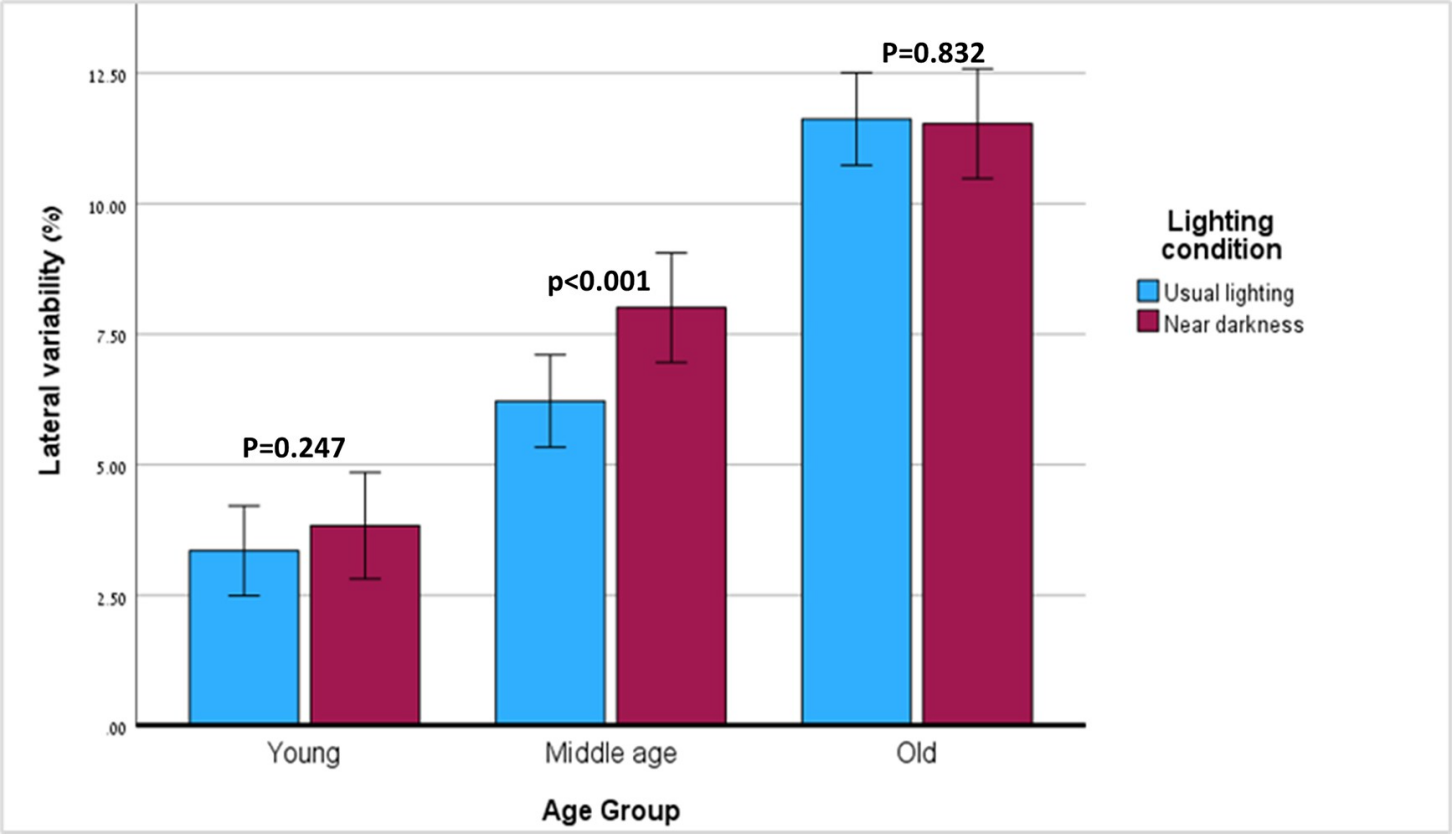
Lateral variability in the two lighting conditions—within-group comparisons.

Within-group comparisons of lateral variability in the three groups under the two lighting conditions is shown in Fig 3. The middle-aged group increased their lateral variability in near-dark compared to usual lighting (6.22, 95% CI: 4.43–8.00 in light versus 8.01, 95% CI: 5.90–10.11 in near-dark, p<0.001, partial η^2^ = 0.505), while the young and the old groups did not change their lateral variability (p = 0.247 and p = 0.832 respectively).

#### Serial -7 walking

There were significant differences between groups in serial-7 performance during walking (p = 0.042, partial η2 = 0.111). The older adults had fewer correct responses compared to the young groups (10.4, 95% CI: 7.2–13.6 vs. 15.3, 95% CI: 13.2–17.5, p = 0.042) but not compared to the middle-aged group (13.8, 95% CI: 10.6–17.0, p = 0.262), and there was no significant difference in serial-7 walking between the middle-aged and young groups (p = 0.262).

## Discussion

The results of the present study show that middle-aged subjects were more affected by the reduced light condition compared with young and old adults. Similar to old adults, middle-aged participants increased their stride time variability when walking in near-darkness, but were the only ones who increased lateral variability and anterior/posterior variability in near-darkness, whereas gait variability of young adults was not affected by the light condition. In addition, middle-aged individuals showed increased lateral variability in both lighting conditions compared with young adults.

Our findings may suggest that age-related changes beginning in middle age impair the ability to regulate gait stability when visual perception is limited due to diminished lighting. Decreased ability of the somatosensory and vestibular systems to compensate for visual input has been suggested as a reason for slow walking speed and decreased postural instability under limited lighting conditions in the elderly population [8,35]. This could also be a possible cause for the increased gait variability observed in the middle-aged group in our study. Indeed, there is evidence that deficits in proprioception or vestibular function may begin in middle age. Hurley et al. [36] and Wingert et al. [37] examined the acuity of joint position sense in young, middle-aged, and elderly subjects and reported age-related deterioration in proprioception. Age-related loss of vestibular function is also common in middle age. A national survey of U.S. adults aged 40 years and older found that the prevalence of vestibular dysfunction was 18.5% in adults aged 40–49 years and increased to 33.0% in adults aged 50–59 years [38,39]. Another possible explanation for the decreased balance during walking in the middle-aged group might be related to the results of the 30CST. While our subjects in all three groups had no walking impairments and were physically active according to their BPAQ score [31,40], the 30CST score in the middle- aged group was below the previously reported age reference value [41] and were similar to those in the old group. Reduced ability to move from sitting to standing has been associated with decreased dynamic stability [42,43]. Therefore, the low 30CST score in our middle-aged group may reflect age-related changes that could affect balance and gait. Overall, human performance in middle age is highly variable [44]. Thus, further research should be conducted to confirm our findings and proposed explanations.

In contrast to our findings, Reynard and Terrier [16] examined gait variability during blindfolded treadmill walking in healthy adults with a mean age of 44±14 years and reported no destabilizing effects on gait. Several explanations can be proposed for this difference. Our subjects were tested with their eyes open in a near-dark environment. It has been shown that it may be more difficult to control gait when walking with eyes open in the dark than when walking with eyes closed (Yelnik et al. 2015). In addition, our subjects were tested under both lighting conditions at their self-selected gait speed measured in normal light, whereas Reynard and Terrier compared gait variability between walking conditions with blindfolded gait speed much slower than normal walking speed. This slower and more careful walking may have allowed subjects to use alternative sensory strategies to control gait dynamics without the aid of vision, so that they did not exhibit impaired stability. Indeed, it has been previously observed that healthy subjects who had been accustomed to reduced light conditions for a sufficiently long time tended to have a near-normal gait (Moe-Nilssen et al. 2006). However, in everyday walking scenarios, there is often a sudden transition from normal to low light or near-darkness, such that the habituation time required to develop a careful gait is insufficient to reduce the challenge of motion control. Finally, while we focused on the middle age, the ages of Reynard and Terrier subjects varied, and their mean age was<45 years. The age difference between subjects and the methods and technologies used to record and analyze gait variability could also explain the different results.

Another study that may not agree with our findings is the study by Kesler et al. [13], which reported that walking in reduced lighting had no effect on the variability of stride and swing time in healthy older people. While Kesler et al. examined overground walking, it is possible that the inherent balance challenges associated with treadmill walking [45] amplified the effects of reduced lighting on stride time variability in the middle-aged and older subjects in our study. In addition, various measures of variability should be measured to assess gait stability [46]. Our study, which assessed both temporal and spatial aspects of gait variability, may improve the understanding of the effects of reduced lighting on gait dynamics. While the middle-aged participants showed increased variability in all three gait outcomes in near darkness, the older adult group did not change their spatial aspects of gait variability (i.e., lateral and anterior/posterior variability) in near darkness. A possible explanation could be related to the high lateral and anterior/posterior variability observed in the older adult group under usual lighting. It is possible that gait stability did not deteriorate further and was less sensitive to the effect of reduced lighting because of the already increased variability values. It should also be noted that our results are consistent with previous data showing higher COP gait variability in middle-aged individuals compared to young adults [20] and the importance of measuring mediolateral dynamic stability outcomes in middle age [19].

Performing an additional task simultaneously with walking did not affect gait stability in either age group under both normal and reduced light conditions. The literature on the effects of cognitive tasks on gait variability in healthy individuals is inconclusive. Some studies show a destabilizing effect, especially in the elderly [47,48], whereas other studies do not [24,49,50]. Our results are more consistent with the latter studies. The type and difficulty of the dual task may influence its effect on gait [51]. Although it has been suggested that walking in near-darkness requires more attention than walking in normal lighting [13], it is possible that the added complexity of walking in near-darkness with counting backward by 7 did not elicit sufficient cognitive load to alter gait stability. Further research is needed to understand why certain cognitive tasks may affect gait variability while other tasks do not.

Although stability during walking was not affected by the additional cognitive task, serial 7 performance of older adults was lower than that of young adults during walking, whereas it did not differ during sitting. This result is consistent with the "posture-first" principle, which has shown that older people prefer to maintain stability when walking while performing another task [52].

This study has several limitations. Our middle-aged group included subjects aged 45 to 65 years. Although middle age is associated with this stage of life, human performance can vary widely during this period. We believe that the results of the present study shed light on the effects of reduced lighting on gait stability in middle age. Nevertheless, future studies with a larger and varied sample may allow analysis of age subgroups within middle age. Another limitation of our study is that it was conducted in an indoor laboratory setting while subjects walked on a treadmill wearing a safety harness. It has already been shown that a non-weight-supporting harness does not alter gait dynamics [53] and that the variability measured during treadmill walking may be an acceptable representation of walking on the ground [54]. However, it is also possible that the effect of reduced lighting would have been greater if subjects had been tested walking overground. Therefore, to raise the ecological validity, it is recommended that future studies also examine the effect of reduced lighting on gait stability in middle-aged adults in an outdoor environment. To further verify our results, it is recommended that such studies will include additional measures of gait stability. Finally, our subjects had no visual impairment or uncorrected problem related to visual acuity. Nevertheless, we did not directly examine aspects of vision as well as other factors, such as vestibular function, that may affect gait. It would also be helpful to investigate these and other relevant aspects in future studies.

## Conclusions

Apart from increased lateral CoP trajectory variability, gait stability in full illumination in middle-age is similar to that of younger people and differs from locomotion in old adults. However, when walking in the dark, a deterioration in balance is mostly pronounced in middle-aged walking. Performing an additional task simultaneously with walking did not affect gait stability in either age group in both normal and reduced light conditions. The results may highlight the need to further investigate postural control in middle-aged adults, as detection of functional deterioration in middle age could promote appropriate interventions that could support better and healthy aging.

## Supporting information

S1 File Appendix 1: Gait variability results. (DOCX)

## References

[1] Patla AE. Understanding the roles of vision in the control of human locomotion. Gait Posture. 1997;5: 54–69. doi: 10.1016/S0966-6362(96)01109-5

[2] Patla AE, Greig M. Any way you look at it, successful obstacle negotiation needs visually guided on-line foot placement regulation during the approach phase. Neurosci Lett. 2006;397: 110–114. doi: 10.1016/j.neulet.2005.12.016 16413969

[3] Perry SD, Santos LC, Patla AE. Contribution of vision and cutaneous sensation to the control of centre of mass (COM) during gait termination. Brain Res. 2001;913: 27–34. doi: 10.1016/s0006-8993(01)02748-2 11532244

[4] Matthis JS, Yates JL, Hayhoe MM. Gaze and the Control of Foot Placement When Walking in Natural Terrain. Curr Biol CB. 2018;28: 1224–1233.e5. doi: 10.1016/j.cub.2018.03.008 29657116 PMC5937949

[5] Rietdyk S, Rhea CK. Control of adaptive locomotion: effect of visual obstruction and visual cues in the environment. Exp Brain Res. 2006;169: 272–278. doi: 10.1007/s00221-005-0345-y 16421728

[6] Moe-Nilssen R, Helbostad JL, Akra T, Birdedal L, Nygaard HA. Modulation of gait during visual adaptation to dark. J Mot Behav. 2006;38: 118–125. doi: 10.3200/JMBR.38.2.118-125 16531394

[7] Saftari LN, Kwon O-S. Ageing vision and falls: a review. J Physiol Anthropol. 2018;37: 11. doi: 10.1186/s40101-018-0170-1 29685171 PMC5913798

[8] Anderson PG, Nienhuis B, Mulder T, Hulstijn W. Are older adults more dependent on visual information in regulating self-motion than younger adults? J Mot Behav. 1998;30: 104–113. doi: 10.1080/00222899809601328 20037026

[9] Huang C-K, Chien J-H, Siu K-C. The reduced lighting environment impacts gait characteristics during walking. Int J Ind Ergon. 2017;61: 126–130. doi: 10.1016/j.ergon.2017.05.005

[10] Freiberger E, Sieber CC, Kob R. Mobility in Older Community-Dwelling Persons: A Narrative Review. Front Physiol. 2020;11. Available: https://www.frontiersin.org/articles/10.3389/fphys.2020.00881. doi: 10.3389/fphys.2020.00881 33041836 PMC7522521

[11] Hausdorff JM. Gait variability: methods, modeling and meaning. J Neuroengineering Rehabil. 2005;2: 19. doi: 10.1186/1743-0003-2-19 16033650 PMC1185560

[12] Figueiro MG, Plitnick B, Rea MS, Gras LZ, Rea MS. Lighting and perceptual cues: Effects on gait measures of older adults at high and low risk for falls. BMC Geriatr. 2011;11: 49. doi: 10.1186/1471-2318-11-49 21864387 PMC3176147

[13] Kesler A, Leibovich G, Herman T, Gruendlinger L, Giladi N, Hausdorff JM. Shedding light on walking in the dark: the effects of reduced lighting on the gait of older adults with a higher-level gait disorder and controls. J NeuroEngineering Rehabil. 2005;2: 27. doi: 10.1186/1743-0003-2-27 16124880 PMC1236955

[14] Wuehr M, Schniepp R, Pradhan C, Ilmberger J, Strupp M, Brandt T, et al. Differential effects of absent visual feedback control on gait variability during different locomotion speeds. Exp Brain Res. 2013;224: 287–294. doi: 10.1007/s00221-012-3310-6 23109084

[15] Saucedo F, Yang F. Effects of visual deprivation on stability among young and older adults during treadmill walking. Gait Posture. 2017;54: 106–111. doi: 10.1016/j.gaitpost.2017.03.001 28284144

[16] Reynard F, Terrier P. Role of visual input in the control of dynamic balance: variability and instability of gait in treadmill walking while blindfolded. Exp Brain Res. 2015;233: 1031–1040. doi: 10.1007/s00221-014-4177-5 25534228

[17] Peeters G, van Schoor NM, Cooper R, Tooth L, Kenny RA. Should prevention of falls start earlier? Co-ordinated analyses of harmonised data on falls in middle-aged adults across four population-based cohort studies. PloS One. 2018;13: e0201989. doi: 10.1371/journal.pone.0201989 30086149 PMC6080796

[18] Peeters G, Cooper R, Tooth L, van Schoor NM, Kenny RA. A comprehensive assessment of risk factors for falls in middle-aged adults: co-ordinated analyses of cohort studies in four countries. Osteoporos Int J Establ Result Coop Eur Found Osteoporos Natl Osteoporos Found USA. 2019;30: 2099–2117. doi: 10.1007/s00198-019-05034-2 31201482

[19] Terrier P, Reynard F. Effect of age on the variability and stability of gait: a cross-sectional treadmill study in healthy individuals between 20 and 69 years of age. Gait Posture. 2015;41: 170–174. doi: 10.1016/j.gaitpost.2014.09.024 25455699

[20] Bizovska L, Svoboda Z, Kutilek P, Janura M, Gaba A, Kovacikova Z. Variability of centre of pressure movement during gait in young and middle-aged women. Gait Posture. 2014;40: 399–402. doi: 10.1016/j.gaitpost.2014.05.065 24973140

[21] Chien JH, Yentes J, Stergiou N, Siu K-C. The Effect of Walking Speed on Gait Variability in Healthy Young, Middle-aged and Elderly Individuals. J Phys Act Nutr Rehabil. 2015;2015: http://www.panr.com.cy/index.php/article/the-effect-of-walking-speed-on-gait-variability-in-healthy-young-middle-aged-and-elderly-individuals/. 26929929 PMC4768759

[22] Muir BC, Haddad JM, van Emmerik REA, Rietdyk S. Changes in the control of obstacle crossing in middle age become evident as gait task difficulty increases. Gait Posture. 2019;70: 254–259. doi: 10.1016/j.gaitpost.2019.01.035 30909004

[23] Yelnik AP, Tasseel Ponche S, Andriantsifanetra C, Provost C, Calvalido A, Rougier P. Walking with eyes closed is easier than walking with eyes open without visual cues: The Romberg task versus the goggle task. Ann Phys Rehabil Med. 2015;58: 332–335. doi: 10.1016/j.rehab.2015.08.004 26439522

[24] Springer S, Giladi N, Peretz C, Yogev G, Simon ES, Hausdorff JM. Dual-tasking effects on gait variability: the role of aging, falls, and executive function. Mov Disord Off J Mov Disord Soc. 2006;21: 950–957. doi: 10.1002/mds.20848 16541455

[25] Yogev G, Hausdorff JM, Giladi N. The Role of Executive Function and Attention in Gait. Mov Disord Off J Mov Disord Soc. 2008;23: 329–472. doi: 10.1002/mds.21720 18058946 PMC2535903

[26] Kearney FC, Harwood RH, Gladman JRF, Lincoln N, Masud T. The relationship between executive function and falls and gait abnormalities in older adults: a systematic review. Dement Geriatr Cogn Disord. 2013;36: 20–35. doi: 10.1159/000350031 23712088

[27] Neider MB, Gaspar JG, McCarley JS, Crowell JA, Kaczmarski H, Kramer AF. Walking & Talking: Dual-Task Effects on Street Crossing Behavior in Older Adults. Psychol Aging. 2011;26: 260. doi: 10.1037/a0021566 21401262 PMC3699858

[28] Yaffe K, Barnes D, Nevitt M, Lui L-Y, Covinsky K. A Prospective Study of Physical Activity and Cognitive Decline in Elderly Women: Women Who Walk. Arch Intern Med. 2001;161: 1703–1708. doi: 10.1001/archinte.161.14.1703 11485502

[29] Atkinson HH, Rapp SR, Williamson JD, Lovato J, Absher JR, Gass M, et al. The Relationship Between Cognitive Function and Physical Performance in Older Women: Results From the Women’s Health Initiative Memory Study. J Gerontol Ser A. 2010;65A: 300–306. doi: 10.1093/gerona/glp149 19789197 PMC2822281

[30] Jones CJ, Rikli RE, Beam WC. A 30-s chair-stand test as a measure of lower body strength in community-residing older adults. Res Q Exerc Sport. 1999;70: 113–119. doi: 10.1080/02701367.1999.10608028 10380242

[31] Baecke JA, Burema J, Frijters JE. A short questionnaire for the measurement of habitual physical activity in epidemiological studies. Am J Clin Nutr. 1982;36: 936–942. doi: 10.1093/ajcn/36.5.936 7137077

[32] Meyer C, Killeen T, Easthope CS, Curt A, Bolliger M, Linnebank M, et al. Familiarization with treadmill walking: How much is enough? Sci Rep. 2019;9: 5232. doi: 10.1038/s41598-019-41721-0 30914746 PMC6435738

[33] Fan Y, Li Z, Han S, Lv C, Zhang B. The influence of gait speed on the stability of walking among the elderly. Gait Posture. 2016;47: 31–36. doi: 10.1016/j.gaitpost.2016.02.018 27264399

[34] Calculating Lakens D. and reporting effect sizes to facilitate cumulative science: a practical primer for t-tests and ANOVAs. Front Psychol. 2013;4. Available: https://www.frontiersin.org/articles/10.3389/fpsyg.2013.00863. 10.3389/fpsyg.2013.00863 PMC3840331 24324449

[35] Anson E, Bigelow RT, Swenor B, Deshpande N, Studenski S, Jeka JJ, et al. Loss of Peripheral Sensory Function Explains Much of the Increase in Postural Sway in Healthy Older Adults. Front Aging Neurosci. 2017;9: 202. doi: 10.3389/fnagi.2017.00202 28676758 PMC5476729

[36] Hurley MV, Rees J, Newham DJ. Quadriceps function, proprioceptive acuity and functional performance in healthy young, middle-aged and elderly subjects. Age Ageing. 1998;27: 55–62. doi: 10.1093/ageing/27.1.55 9504367

[37] Wingert JR, Welder C, Foo P. Age-related hip proprioception declines: effects on postural sway and dynamic balance. Arch Phys Med Rehabil. 2014;95: 253–261. doi: 10.1016/j.apmr.2013.08.012 23994251

[38] Allen D, Ribeiro L, Arshad Q, Seemungal BM. Age-Related Vestibular Loss: Current Understanding and Future Research Directions. Front Neurol. 2016;7: 231. doi: 10.3389/fneur.2016.00231 28066316 PMC5165261

[39] Agrawal Y, Carey JP, Della Santina CC, Schubert MC, Minor LB. Disorders of Balance and Vestibular Function in US Adults: Data From the National Health and Nutrition Examination Survey, 2001–2004. Arch Intern Med. 2009;169: 938–944. doi: 10.1001/archinternmed.2009.66 19468085

[40] Tebar WR, Ritti-Dias RM, Fernandes RA, Damato TMM, de Barros MVG, Mota J, et al. Validity and reliability of the Baecke questionnaire against accelerometer-measured physical activity in community dwelling adults according to educational level. PLoS ONE. 2022;17: e0270265. doi: 10.1371/journal.pone.0270265 35969609 PMC9377570

[41] Tveter AT, Dagfinrud H, Moseng T, Holm I. Health-related physical fitness measures: reference values and reference equations for use in clinical practice. Arch Phys Med Rehabil. 2014;95: 1366–1373. doi: 10.1016/j.apmr.2014.02.016 24607837

[42] Yee XS, Ng YS, Allen JC, Latib A, Tay EL, Abu Bakar HM, et al. Performance on sit-to-stand tests in relation to measures of functional fitness and sarcopenia diagnosis in community-dwelling older adults. Eur Rev Aging Phys Act. 2021;18: 1. doi: 10.1186/s11556-020-00255-5 33419399 PMC7791746

[43] Whitney SL, Wrisley DM, Marchetti GF, Gee MA, Redfern MS, Furman JM. Clinical Measurement of Sit-to-Stand Performance in People With Balance Disorders: Validity of Data for the Five-Times-Sit-to-Stand Test. Phys Ther. 2005;85: 1034–1045. doi: 10.1093/ptj/85.10.1034 16180952

[44] Hunter SK, Pereira HM, Keenan KG. The aging neuromuscular system and motor performance. J Appl Physiol. 2016;121: 982–995. doi: 10.1152/japplphysiol.00475.2016 27516536 PMC5142309

[45] Yang F, King GA. Dynamic gait stability of treadmill versus overground walking in young adults. J Electromyogr Kinesiol. 2016;31: 81–87. doi: 10.1016/j.jelekin.2016.09.004 27694060

[46] Moe-Nilssen R, Aaslund MK, Hodt-Billington C, Helbostad JL. Gait variability measures may represent different constructs. Gait Posture. 2010;32: 98–101. doi: 10.1016/j.gaitpost.2010.03.019 20434916

[47] Dubost V, Kressig RW, Gonthier R, Herrmann FR, Aminian K, Najafi B, et al. Relationships between dual-task related changes in stride velocity and stride time variability in healthy older adults. Hum Mov Sci. 2006;25: 372–382. doi: 10.1016/j.humov.2006.03.004 16714067

[48] Hollman JH, Kovash FM, Kubik JJ, Linbo RA. Age-related differences in spatiotemporal markers of gait stability during dual task walking. Gait Posture. 2007;26: 113–119. doi: 10.1016/j.gaitpost.2006.08.005 16959488

[49] Yogev G, Giladi N, Peretz C, Springer S, Simon ES, Hausdorff JM. Dual tasking, gait rhythmicity, and Parkinson’s disease: which aspects of gait are attention demanding? Eur J Neurosci. 2005;22: 1248–1256. doi: 10.1111/j.1460-9568.2005.04298.x 16176368

[50] Zadik S, Benady A, Gutwillig S, Florentine MM, Solymani RE, Plotnik M. Age related changes in gait variability, asymmetry, and bilateral coordination—When does deterioration starts? Gait Posture. 2022;96: 87–92. doi: 10.1016/j.gaitpost.2022.05.009 35617787

[51] Goh H-T, Pearce M, Vas A. Task matters: an investigation on the effect of different secondary tasks on dual-task gait in older adults. BMC Geriatr. 2021;21: 510. doi: 10.1186/s12877-021-02464-8 34563129 PMC8465774

[52] Lion A, Spada RS, Bosser G, Gauchard GC, Anello G, Bosco P, et al. “Postural first” principle when balance is challenged in elderly people. Int J Neurosci. 2014;124: 558–566. doi: 10.3109/00207454.2013.864288 24205810

[53] Stout RD, Wittstein MW, LoJacono CT, Rhea CK. Gait dynamics when wearing a treadmill safety harness. Gait Posture. 2016;44: 100–102. doi: 10.1016/j.gaitpost.2015.11.012 27004640

[54] Owings TM, Grabiner MD. Step width variability, but not step length variability or step time variability, discriminates gait of healthy young and older adults during treadmill locomotion. J Biomech. 2004;37: 935–938. doi: 10.1016/j.jbiomech.2003.11.012 15111081

