# Supplementary material for: Young, but not in the dark—The influence of reduced lighting on gait stability in middle-aged adults
Source: PLoS One. 2023 May 18;18(5):e0280535. doi: 10.1371/journal.pone.0280535 (PMC10194872; doi:10.1371/journal.pone.0280535)
Supplement: S1 File — (DOCX) [file pone.0280535.s001.docx]

**Appendix 1: Gait variability results**

| **Old**  **N=19** | **Middle age**  **N=20** | **Young**  **N=20** |  | |
| --- | --- | --- | --- | --- |
| 4.09±1.07*^#^  (3.49-4.70) | 2.32±1.07  (1.73-2.90) | 1.71±1.07  (1.13-2.29) | All walking conditions | **Stride time variability** |
| 4.56±1.31*^#^  (3.82-5.29) | 2.84±1.31  (2.13-3.55) | 1.91±1.31  (1.20-2.62) | Near-darkness |  |
| 3.63±0.94*^#^  (3.10-4.16) | 1.79±0.95  (1.28-2.30) | 1.52±0.95  (1.01-2.03) | Usual lighting |  |
| 6.37±1.60*  (5.59-7.15) | 4.36±1.60  (3.55-5.16) | 3.95±1.60  (3.19-4.71) | All walking conditions | **Anterior/posterior variability** |
| 6.49±1.92*^#^  (5.56-7.43) | 5.02±1.92  (4.05-5.98) | 4.15±1.91  (3.25-5.06) | Near-darkness |  |
| 6.25±1.68*^#^  (5.43-7.07) | 3.69±1.68  (2.85-4.54) | 3.75±1.68  (2.95-4.55) | Usual lighting |  |
| 11.58±3.91*^#^  (9.67-13.48) | 7.11±3.91*  (5.21-9.02) | 3.59±3.91  (1.74-5.44) | All walking conditions | **Lateral variability** |
| 11.53±4.33*^#^  (9.42-13.64) | 8.01±4.33*  (5.90-10.12) | 3.83±4.32^#^  (1.78-5.88) | Near-darkness |  |
| 11.62±3.65*^#^  (9.84-13.40) | 6.22±3.65*  (4.44-8.00) | 3.35±3.65^#^  (1.62-5.08) | Usual lighting |  |

*Values ​​are presented as mean ±SD (95% confidence interval)*

** Significant difference compared to young*

*^#^ Significant difference compared to middle age*
