# Supplementary material for: Young, but not in the dark—The influence of reduced lighting on gait stability in middle-aged adults
Source: PLoS One. 2023 May 18;18(5):e0280535. doi: 10.1371/journal.pone.0280535 (PMC10194872; doi:10.1371/journal.pone.0280535)
Supplement: Attachment — Submitted filename: PLoSONE_Review_JAN23.docx [file pone.0280535.s002.docx]

Journal: PLOS One

Manuscript #: PONE-D-22-35579

Title: Young, but not in the Dark - the Influence of Reduced Lighting on Gait Stability in

Middle-aged adults

General comments about the manuscript:

Reject

The authors present a study of the effects of dual task and environmental light levels on gait stability during treadmill walking in young, middle-aged, and older adults. I have presented a list of major and minor concerns below that can make the manuscript a better paper and more suitable for publication, however, it is my professional opinion that these data are not suitable for publication in PLOS One. The authors should consider publication in more gait and posture specific journals.

**Major Concerns**

Methods:

- Were subjects matched in any way (sex? Concussion history?)
  - Authors should consider concussion history if possible in sensitivity anlayses
- Any concern that the dim lighting didn’t affect gait as much as hypothesized due to walking on the treadmill and not overground? Please elaborate in the limitations section.
- Due to group differences in baseline demographics that may affect gait, it would behoove the authors to consider a sensitivity analysis comparing groups when controlling for these factors using an ANCOVA.

Results/Discussion:

- Failure to account for the below normal physical abilities of the middle-aged group may have biased your results and thus clinical interpretations of the data. I would reanalyze the data with control for the confounders, and re-write these sections accordingly.

**Minor Concerns**

Abstract:

- Try to use key words that aren’t in the title to increase searchability of the manuscript (e.g., older adults, walking, visual system, sensory reorganization)
- Include effect sizes for results

Methods:

- L90-95: These assessments come out of nowhere, and therefore an argument for their inclusion should be made in the introduction, not in the methods section.
- Include statement on effect sizes interpretations with citation

Results:

- Very wordy, see comment below on Table 2 as a means to make this section more concise.

Tables:

- Table 1: Include effect sizes
- Table 2: Can likely add p-values/effect sizes here to eliminate the lengthy text in the results section. Also consider limiting to 2 decimal places

Figutres:

- Please reupload Figures 1-3 as they are blurry. It may benefit the reader as well if the authors show individual data points or a violin plot with boxplot overlay instead of a bar chart to better highlight individual data points.
- Figures 1-3 are Table 2 represented visually, pick one or the other as they are repetitive. My recommendation would be to keep the figures, remove the tables, but include the descriptions/numbers in the result section.

Discussion

- Should add regarding lack of differences in the older adult group

References:
