# Supplementary material for: Young, but not in the dark—The influence of reduced lighting on gait stability in middle-aged adults
Source: PLoS One. 2023 May 18;18(5):e0280535. doi: 10.1371/journal.pone.0280535 (PMC10194872; doi:10.1371/journal.pone.0280535)
Supplement: Attachment — Submitted filename: Response to Reviewers.docx [file pone.0280535.s004.docx]

Response to the Comments of the Reviewers on Manuscript ID PONE-D-22-35579R1

We thank the reviewers and the editor for the opportunity to consider a revised version of this manuscript. Both reviewers have indicated that we have addressed all of the comments and concerns they raised and have recommended that our paper be accepted, subject to the minor revision suggested by reviewer #1. We present that comment and our response below.

Reviewer #1:

Authors did a sufficient job incorporating reviewer concerns. My only concern is the removal of post-hoc and stating "planned comparisons" multiple times throughout the manuscripts make it seem like it wasn't planned due to the excessive nature of restating that it was planned (most things in research are already planned, so why specify so much?). Otherwise, happy with the submission!

**Response:** The reviewer is right and we thank the reviewer for this comment. Based on this comment, we have reduced the multiple mentions of "planned comparisons" throughout the manuscript.
